# Toward schistosomiasis control: Assessment of infection-associated voiding symptoms, quality of life and the impact of exercise coupled with water intake on egg recovery in an endemic community in Ghana

**DOI:** 10.1371/journal.pgph.0002514

**Published:** 2023-11-20

**Authors:** Samuel Essien-Baidoo, Mainprice Akuoko Essuman, Joseph Tee, Richard K. D. Ephraim, Loretta Betty Blay Mensah, Seth Boakye Amponsah, Justice Afrifa

**Affiliations:** Department of Medical Laboratory Science, School of Allied Health Science, College of Health and Allied Sciences, University of Cape Coast, Cape Coast, Ghana; Kamuzu University of Health Sciences, MALAWI

## Abstract

Assessment of the burden of disease and techniques for clinical diagnosis could ultimately help in schistosomiasis control. This study assessed the impact of exercises and water intake on ova recovery during laboratory diagnosis and schistosomiasis-associated urinary symptoms and quality of life (QOL) among inhabitants of Dendo, an endemic community in Ghana. The clinical findings and responses of 400 randomly selected participants were used for the study. The International Prostate Symptoms Score (I-PSS) was used to collect information on participants’ self-reported urinary symptoms and QOL. Finally, urine samples were collected on two consecutive days, initially without exercise and water intake and then after exercise and water intake, and about 10 ml of it were microscopically examined for the presence and quantification of ova. The data collected from the study were analyzed using IBM SPSS. *Schistosoma haematobium* egg recovery increased significantly (p < 0.001) from 206 (51.5%) to 220 (55.0%) after exercise and water intake with the highest increase being observed among participants less than 20 years (53.3% to 57.1% after exercise and water intake). As high as 90.3% and 56.8% of Schistosoma-positive participants reported IPSS>7 (symptomatic voiding disorders) and QOL≥4 (mostly dissatisfied or unhappy QOL) respectively. The commonest voiding symptoms reported were nocturia (98.9%) and incomplete emptying (79.6%). Positive correlations between egg count, IPSS score, and QOL were observed. This study provides important evidence for the inclusion of exercise and water intake in the microscopic diagnosis of *Schistosoma haematobium* and reveals that schistosomiasis significantly impacts the affected individuals’ urinary health and overall quality of life.

## 1. Introduction

Schistosomiasis, also known as bilharzia, is one of the major parasitic infections in the world, especially in poverty-driven areas with poor sanitary conditions [1]. Urogenital schistosomiasis caused by *Schistosoma haematobium* accounts for approximately two-thirds of most parasitic infections [2]. Over 250 million people worldwide are infected and approximately 85% of the infected persons live in sub-Saharan Africa [3, 4].

Public awareness and concern for schistosomiasis in Ghana go back to the 1960s during the construction of the Akosombo dam. The construction of the dam resulted in the sudden occurrence and infestation of *Bulinus truncatus* (an intermediate host of *S*. *haematobium*), resulting in urogenital schistosomiasis in several communities along the lake [5]. This situation was compounded after the construction of the Kpong dam [6]. Today, Ghana is considered endemic to Schistosomiasis caused by *S*. *haematobium* and *S*. *mansoni* and is cited to be present throughout the whole country including urban areas according to the 2015 World Schistosomiasis Risk Chart [7]. Various surveys recently carried out across the country have reported prevalence ranging from 12.8–66.8% for urinary schistosomiasis caused by *S*. *haematobium* and 11.9%-90.5 for intestinal schistosomiasis caused by *S*. *mansoni* [8–13].

In urinary Schistosomiasis, terminal-spined eggs enter tissues and are expelled in urine. For this to occur, female *S*. *haematobium* pairs, which are present in the veins draining important pelvic organs including the bladder, uterus, and cervix, must be present. However, many *S*. *haematobium* eggs fail to exit the body and dislodge within the capillary beds of pelvic end-organs, especially the tissues of the bladder, ureters, and genital organs causing granulomas and ultimately small fibrotic nodules [14]. This leads to life-threatening disorders such as haematuria, bladder cancer, ureteric blockage, hydronephrosis, urinary tract infections, renal failure, and in some cases death [15]. Consequently, a lot of *S*. *haematobium*-infected persons have complained that infection affects their quality of life [16, 17]. Although pathologic lesions in the bladder and genitourinary system have been reported [18–21] in *S*. *haematobium* infections, disruptions in voiding have received relatively little attention, and there are rare reports describing the voiding function of infected individuals. Symptoms and Health-Related Quality of Life (HRQOL) assessment, along with morbidity and mortality measurements, are critical for determining disease burden, evaluating interventions and standard healthcare policies, and identifying areas for further improvement [22].

Even though the infection is treatable, early detection of the parasites is ideal for the prevention of possible sequelae into related health problems. In advanced countries, more sensitive and specific techniques such as antigen, antibody, and DNA detection techniques are used in *S*. *haematobium* diagnosis [4]. In developing countries like Ghana, the traditional diagnostic procedure used in most settings is the detection and quantification of the parasites’ ova that is shed into urine by light microscopy [23]. In line with the World Health Organization (WHO) recommendation [24], patients are usually encouraged to engage in short physical exercises (such as jumping or running) to help in the detachment of the ova from the walls of the bladder into the urine. This recommendation was proposed based on an earlier report that physical exercises together with the intake of fluid before micturition could increase egg output significantly [25]. However, very limited evidence is available on the effectiveness of this recommendation. To date, only a single study has evaluated the efficacy of this practice for *S*. *haematobium* diagnosis [26]. In the study, egg excretion did not increase with exercises before micturition, but the authors proposed further studies to authenticate this claim. Validation of this recommendation would go a long way to provide empirical evidence for acceptance and formalization of the practice, knowing that it impacts the sensitivity of microscopy in *S*. *haematobium* diagnosis. There is a need to further validate this practice in different populations.

This study, therefore, aimed to evaluate the effect of simple exercises combined with water intake on *S*. *haematobium* ova recovery during microscopic examination. Again, the International Prostate Symptom Score (IPSS) questionnaire was used to evaluate urinary symptoms and QOL associated with *S*. *haematobium* infection in an endemic community in Ghana.

## 2. Methods

### 2.1. Study design

This study was designed as a cross-sectional study to evaluate the impact of simple exercises and water intake on the microscopic diagnosis of urinary schistosomiasis and to assess the effect of *S*. *haematobium* infection on urinary health and quality of life.

### 2.2. Study site

**Fig 1:**
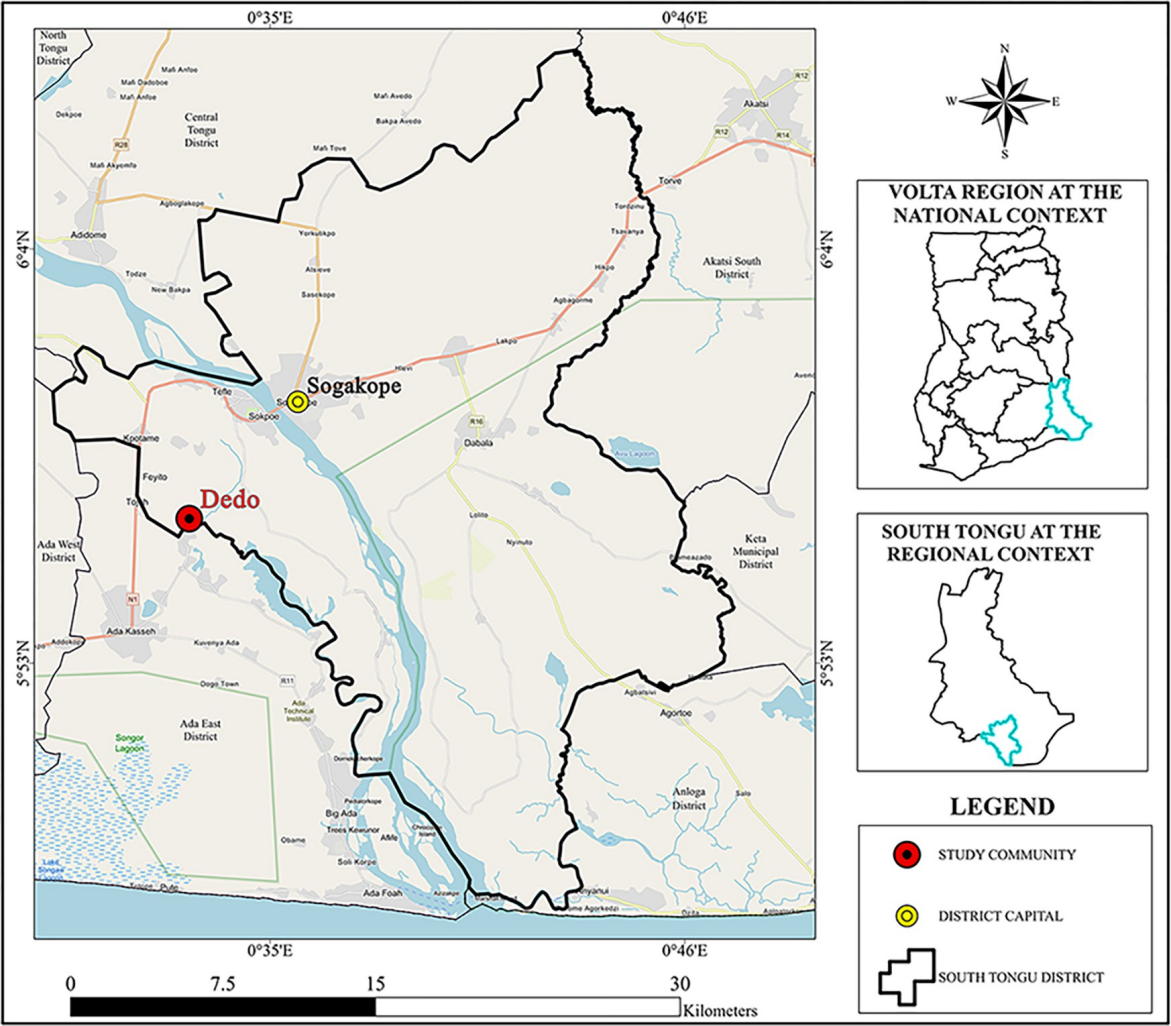
Map of South Tongu District showing the location of the Dendo community. The basemap shapefile was obtained from the Cartography and GIS Unit, Department of Geography and Regional Planning, University of Cape Coast, Ghana. The map was developed using ArcGIS software version 10.2.

The study was conducted at Dendo (Fig 1), a community in the South Tongu District of the Volta Region of Ghana. According to the 2010 Population and Housing Census [27], South Tongu District has 87,950 representing 4.1% of the total population of the Volta Region. Approximately, 54.5% of inhabitants are females with 45.5% being males. Dendo has an approximate population of 1,071, comprising 560 females and 511 males. About 569 (53.13%) of the inhabitants of Dendo are less than 20 years old. The major occupation of the people is crop and fish farming, and craftsmanship with only a few in white-color jobs. The main source of water for the community is the Angor River situated less than 500 meters from the community. A variable urinary schistosomiasis prevalence of 2.5% [28], 26.0% [29], and 10.4% [30] has earlier been reported in the district.

### 2.3. Sample size and selection

The minimum sample size required for the study was determined using the StatCalc function of the Epi Info software (Version 7.2.5.0, Centers for Disease Control and Prevention, Atlanta, GA 30333). The following assumptions were made: 95% Confidence interval, 5% acceptable margin of error, and prevalence of 26% based on prevalence at Sogakope–a nearby community [29]. The sample size was inflated by 30% to cater for non-response and increase the study’s power. A total of 400 consenting participants were recruited for the study. In each household in the community, simple random sampling using the lottery method was used to recruit consenting individuals. Participants were required to be: (i) residents of the Dendo community, (ii) not have serious chronic disorders, and (iii) willing to participate in the study. Individuals who failed to give consent had observable chronic disorders and those who were not staying in the Dendo community were excluded from the study.

### 2.4. Questionnaire

A questionnaire was administered to participants to provide their ages and gender. The questionnaire also contained items that allowed participants to self-report their quality of life and voiding symptoms with the assistance of one of the researchers who could translate the study items. The items for this section were obtained from the International Prostate Symptoms Score (IPSS) which is formally known as the American Urological Association (AUA) symptom index (S1 Text).

### 2.5. Urine sample collection

For parasitological examination, two urine samples were collected from participants between 10:00 a.m. and 2:00 p.m. on two consecutive days at their homes on the days of collection. Each participant was given sterile leak-proof plastic containers labeled with a unique identification number to provide about 50ml mid-stream urine. For all participants, samples were provided without exercise on the first day. On the second day, participants were made to undergo simple exercises like running and jumping for 5–10 minutes and to take 300–500 ml of water before micturition. In both days, a member of the team was in attendance to ensure that participants adhered to instructions for engaging in the exercises, taking water and urine collection. Urine samples were kept at ambient temperature between the collection sites and the laboratory for parasitological examination on the same day.

### 2.6. Parasitological examination

Urine parasitological examination by urine sedimentation and light microscopy was conducted following standard protocols as used in an earlier study [10]. The intensity of infection was classified as mild (≤ 15 eggs/10 ml of urine), moderate (16–50 eggs/10 ml of urine), or severe (≥ 50 eggs/10 ml of urine) [31].

### 2.7. Data analysis

The data obtained were entered into Microsoft Excel (Microsoft Windows, version 2016), double-checked, and analyzed with IBM SPSS (version 26) and GraphPad Prism (version 8) software packages. Descriptive statistics such as frequencies, percentages, and means were used to summarize the background characteristics and other data for the study. The Chi-square analysis, Fisher’s exact test, and Mann–Whiney U-test were used to explore differences between groups. The association between age, egg count, IPSS score, and QOL score was explored using correlation analysis and presented using scatter plots. All statistical analyses were two-tailed with the level of significance set to P <0.05.

### 2.8. Ethical consideration

The Declaration of Helsinki and local regulatory requirements were followed in conducting this study. The respondents were provided with the full details of the study and made to provide verbal informed consent before being enrolled. For children less than 18 years old, consent was obtained from their parents or guardians. Participants’ information was strictly kept anonymous and confidential. Infected participants and those with moderate to severe urinary symptoms were counseled and referred to the District Hospital for appropriate clinical attention.

## 3. Results

### 3.1. Age and gender composition of study participants

The majority of the participants were females (53.3%) and were also less than 20 years old (84.0%) **(Table 1)**. The mean age of the study participants was 15.1 years and ranged from 5 years to 78 years. The average ages of females and males were 16.15±14.35 and 13.98±10.70 respectively.

**Table 1:** Age and gender composition of study participants.

Characteristic	Number (%) of participants who participated in the survey		
	Female	Male	Total
No. of respondents	213 (53.3)	187 (46.8)	400 (100.0)
Age			
Mean	16.2	14.0	15.1
Range	5–78	5–65	5–78
Age groups			
< 20	175 (82.2)	161 (86.1)	336 (84.0)
20–39	17 (8.0)	17 (9.1)	34 (8.5)
40–59	13 (6.1)	7 (3.7)	20 (5.0)
60+	8 (3.8)	2 (1.1)	10 (2.5)

### 3.2. Effect of exercise and intake of water on *S*. *haematobium* egg recovery

**Table 2** details the rate of egg recovery from urine samples categorized based on age and infection intensity. Overall, *S*. *haematobium* egg recovery increased significantly (p < 0.001) from 206 (51.5%) in urine collected without exercising on day one to 220 (55.0%) for urine samples collected after exercising on day two. The greatest egg recovery was observed among participants less than 20 years, as egg recovery increased from 179/336 (53.3%) without exercise to 192/336 (57.1%) after exercising. Among participants aged 40 and above, exercise did not cause any change in egg recovery in urine. The number of participants with severe infection (>50 ova/ 10ml) after exercise increased across all the age groups after exercising.

**Table 2:** The distribution of *S*. *haematobium* infection before and after exercise was stratified by age.

	Age categories									
	< 20		20–39		40–59		60+		Total	
	Day one	Day two	Day one	Day two	Day one	Day two	Day one	Day two	Day one	Day two
Mean egg concentration (Eggs per 10 mL of urine)	5.02 ± 6.48	21.25 ± 27.02	4.00 ± 6.02	12.50 ± 21.04	1.95 ± 3.49	9.00 ± 17.49	4.40 ± 6.40	9.40 ± 17.92	4.77 ± 6.35	19.60 ± 26.20
Infection intensity										
No infection (0)	157 (46.7)	144 (42.9)	20 (58.8)	19 (55.9)	12 (60.0)	12 (60.0)	5 (50.0)	5 (50.0)	194 (48.5)	180 (45.0)
Low (1–15)	155 (46.1)	72 (21.4)	12 (35.3)	7 (20.6)	8 (40.0)	5 (25.0)	4 (40.0)	4 (40.0)	179 (44.8)	88 (22.0)
Moderate (16–50)	24 (7.1)	14 (4.2)	2 (5.9)	2 (5.9)	0 (0.0)	1 (5.0)	1 (10.0)	0 (0.0)	27 (6.8)	17 (4.3)
Heavy (>50)	0 (0.0)	106 (31.5)	0 (0.0)	6 (17.6)	0 (0.0)	2 (10.0)	0 (0.0)	1 (10.0)	0 (0.0)	115 (28.8)

On day one urine samples were taken without participants engaging in exercises and taking water. On day two urine samples were taken after participants had engaged in exercises and taken water.

**Fig 2:**
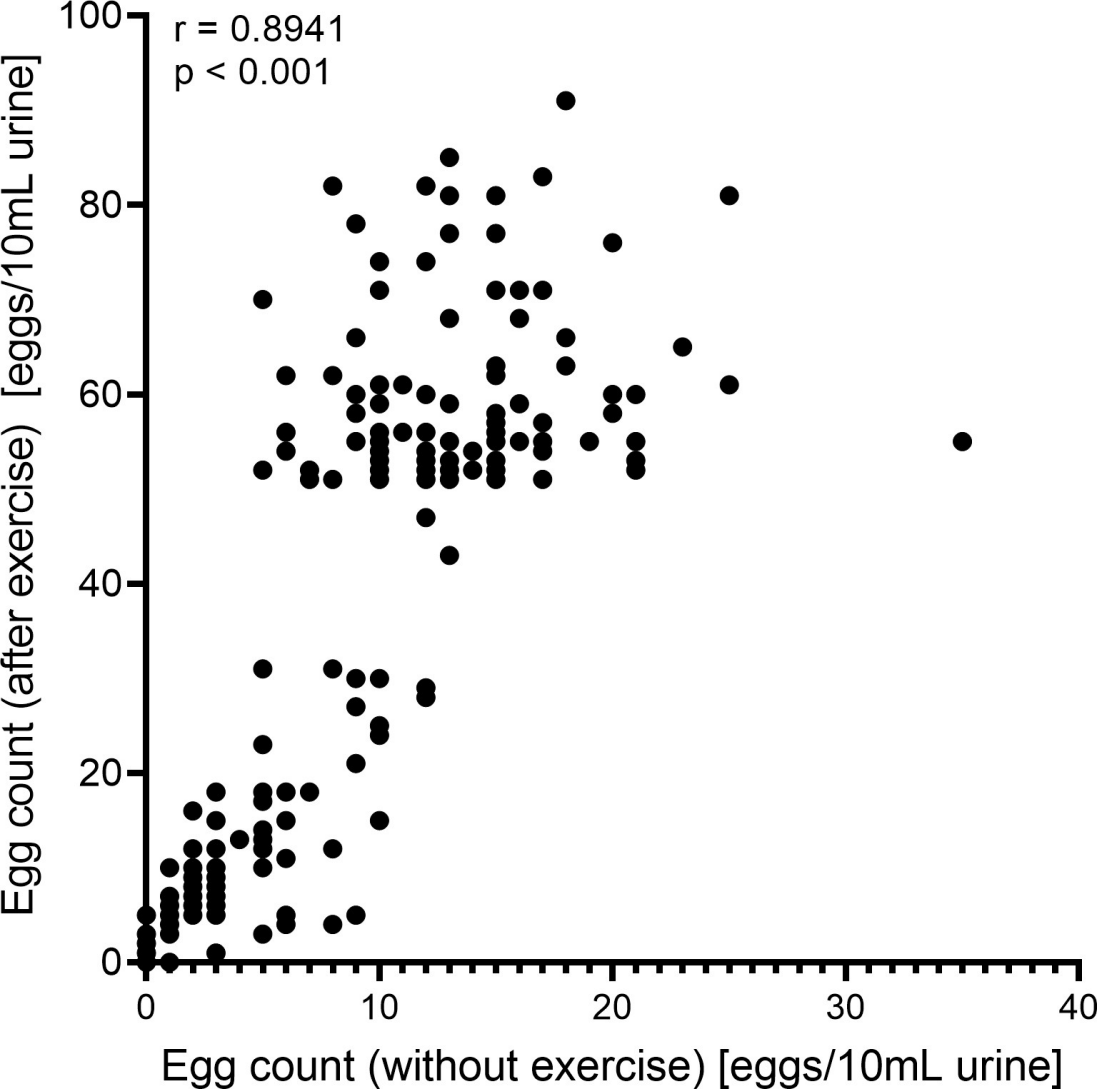
Association between egg count on day one (without exercise) and day two (after exercise and water intake) based on Pearson’s correlation.

We found a strong positive correlation between egg count for urine collected without exercise and after exercise **(Fig 2).**

### 3.3. Impact of The *S*. *haematobium* infection on urinary voiding symptoms and quality of life

The distribution of scores on the IPSS for each of the voiding symptoms is detailed in **Table 3**. Among the symptoms, nocturia (98.9%) and incomplete emptying (79.6%) had the highest number of participants scoring ≥1.

**Table 3:** Distribution of individual voiding symptom scores reported by respondents.

Symptoms	Distribution of IPSS, %						% with ≥1 score
	0	1	2	3	4	5	
Voiding symptoms							
Incomplete Emptying	20.5	34	14	9.3	7.5	14.8	79.6
Frequency	31.3	29.5	15.8	13.3	6.5	3.8	68.9
Intermittency	23.8	32.3	18.8	11.5	6.3	7.5	76.4
Urgency	27.8	30.5	16.5	9.3	6	10	72.3
Weak stream	27.3	34.3	18.5	13	4.5	2.5	72.8
Straining	25.8	29.5	15.5	8.3	6	15	74.3
Nocturia	1.3	11.8	54	16.3	11.5	5.3	98.9
Quality of Life	6.8	39.5	13.8	10.3	2.5	27.3	93.4

**Table 4** details the distribution of participants reporting IPSS scores >7 (symptomatic voiding symptoms) and the average scores for each of the voiding symptoms. The average overall IPSS score in Schistosoma-positive participants was significantly higher than in those without Schistosoma (19.02±7.97 vs 5.30±1.73, p < 0.001). Schistosoma-positive participants reported significantly higher scores in all urinary voiding symptoms when compared to Schistosoma-negative participants.

**Table 4:** Comparison of IPSS and QOL scores among participants with and without schistosomiasis.

Study items	Total	Negative	Positive	P-value
Mean score of IPSS				
Incomplete emptying	1.94±1.70	0.66±0.60	3.13±1.52	<0.001
Frequency	1.46±1.41	0.49±0.58	2.36±1.36	<0.001
Intermittency	1.67±1.49	0.59±0.57	2.68±1.37	<0.001
Urgency	1.65±1.59	0.57±0.63	2.67±1.55	<0.001
Weak stream	1.41±1.27	0.56±0.55	2.21±1.24	<0.001
Straining	1.84±1.73	0.61±0.61	3.00±1.64	<0.001
Nocturia	2.41±1.05	1.81±0.46	2.97±1.13	<0.001
Total IPSS score	12.37±9.01	5.30±1.73	19.02±7.97	<0.001
Mean quality of life score	2.44±1.77	1.11±0.69	3.69±1.56	<0.001
Number (%) with IPSS >7	206 (51.5)	20 (10.3)	186 (90.3)	<0.001
Number (%) with QoL >3	119 (29.8)	2 (1.0)	117 (56.8)	<0.001

Regarding participants’ quality of life, over 90% of participants reported a score ≥1 with an overall average score of 2.44±1.77. Schistosoma-positive participants reported a higher score on the QOL scale compared to Schistosoma-negative participants (3.69±1.56 vs 1.11±0.69, p < 0.001). As high as 56.8% of Schistosoma-positive participants reported QOL >3 as against only 1.0% of Schistosoma-negative participants.

### 3.4. Associations between age, egg count, IPSS score, and QOL score

**Fig 3:**
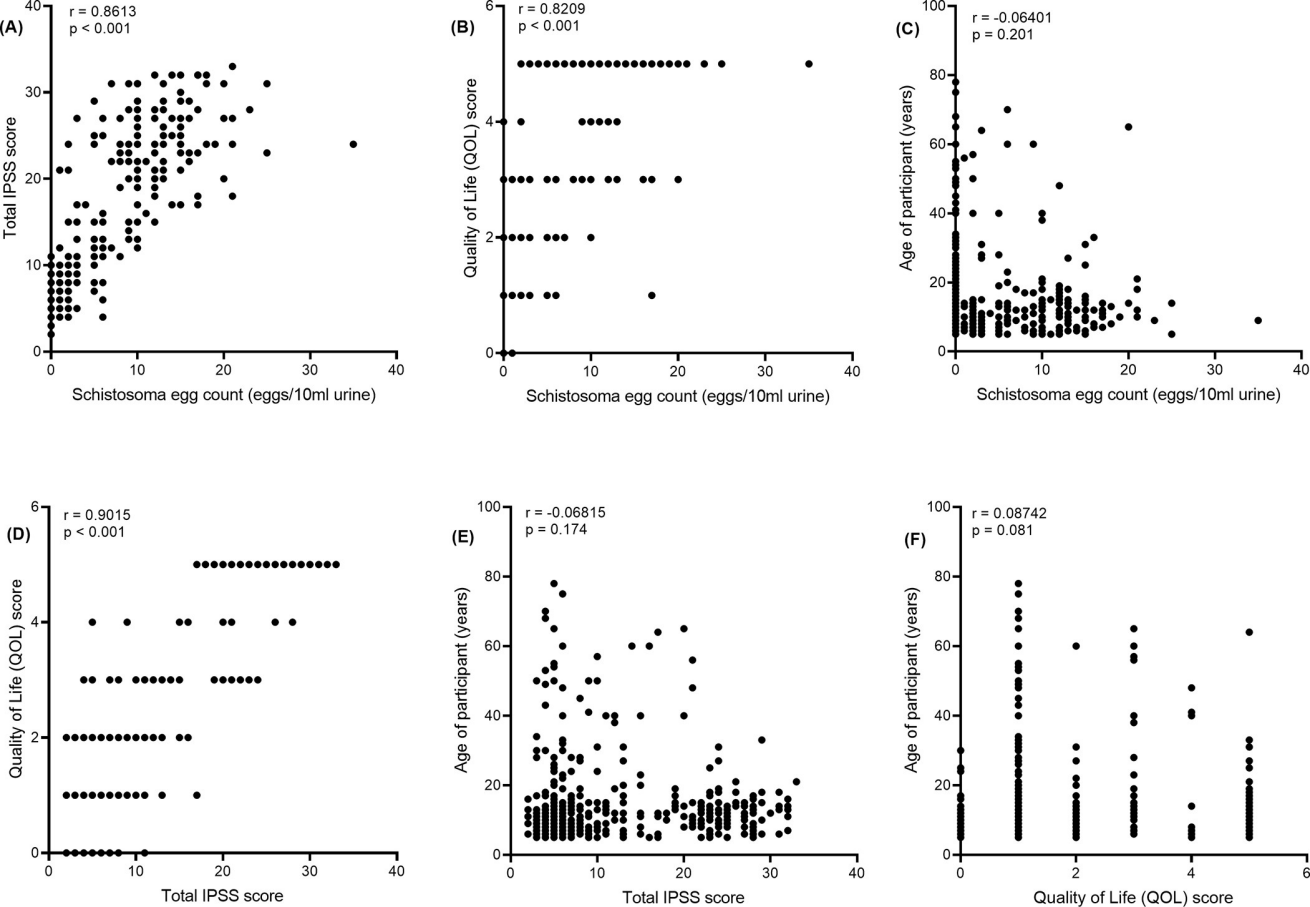
Association between (A) egg count and IPSS score, (B) egg count and QOL score, (C) egg count and age, (D) IPSS score and QOL score, (E) IPSS score and age, and (F) QOL score and age based on Pearson’s correlation.

We found a strong correlation between IPSS score and egg count, IPSS score and QOL score, egg count and QOL score among participants. However, there was no significant association between age and either egg count, I-PSS score, or QOL score (**Fig 3**).

## 4. Discussion

This study evaluated the utility of simple exercises and fluid intake as recommended by the WHO for microscopic diagnosis of urinary schistosomiasis. Again, the study evaluated the burden of *S*. *haematobium*-associated urinary symptoms among people living in an endemic community in Ghana. There was a relatively high recovery of *S*. *haematobium* ova among 51.5% of participants even without exercise. This finding reflects the burden of disease in the Region possibly due to over-reliance on the Angor and Volta Rivers for water, swimming, farming, and domestic activities. Earlier, urinary schistosomiasis prevalence of 26% in Sogakope [29], and 10.36% in Adaklu and Agotime-Ziope districts [30], all in the Region have been reported. It must be mentioned that this level of prevalence is relatively lower than that recently recorded in other parts of the country [11, 32]. For about two years before the study, there had not been any form of deworming exercise in the district possibly due to attention given to and limitations caused by the COVID-19 pandemic.

The urine microscopy technique commonly used in *S*. *haematobium* diagnosis has been fraught with some concerns. The extent to which the presence and intensity of discharged eggs in urine is related to the real degree of genital morbidity is still up for discussion. Studies have indicated that a significant fraction of adult women with vaginal lesions as a result of female genital schistosomiasis were found to be urine microscopy negative [33]. Thus, the need to explore techniques that could help improve the utility of microscopy such as the WHO’s recommendation to include exercise and fluid intake. The present study reports a significant increase in egg recovery on the second day when participants were made to engage in simple exercises and given water before micturition. This finding corroborates that reported earlier [25] which occasioned WHO’s recommendation to exercise before micturition [24] but is different from a recent study by Coulibaly, Andrews [26]. Contrary to WHO’s recommendation and Doehring, Feldmeier [25]’s protocol, Coulibaly, Andrews [26]’s study did not include the intake of water by participants. This was therefore stated as a possible reason for a contrary observation. In urinary schistosomiasis, the venules around the pelvic organs serve as a home for adult worms where they lay their eggs [34]. In urinary excretion, some of these eggs are trapped in the bladder mucosa [34, 35]. It is reported that in excreting *S*. *haematobium* eggs through urine, about 75% of females may have eggs stacked in their uterus, cervix, vagina, or vulva [33]. Although the direct role of exercise and water intake is not known, exercise may facilitate the mechanical expulsion of these eggs for their detection in urine.

Knowledge of the true burden of disease could potentially help in disease diagnosis and management. However, the impact of *S*. *haematobium* infection on voiding symptoms has received little attention. In the present study, an increase in voiding symptoms strongly associated with infection intensity was observed among *S*. *haematobium*-infected participants. This observation deviates from an earlier report among school children in Kenya [36]. While the study attributed this observation partly to the age of participants, the present study did not find any association between the IPSS score and age. The urinary symptoms may be caused by a host of factors including inflammation, bladder obstruction and reactivity, tumors, infections, and neuropathies [37–39] most of which are characteristic of urinary schistosomiasis [40].

The commonest symptomatic voiding disorders reported were nocturia (98.9%) and incomplete emptying. Aside from the inconvenience it creates, nocturia which may be caused by bladder reactivity and storage disorders can result in sleep deprivation which ultimately impedes health and daytime functioning [39]. These symptoms come together to affect the quality of life of individuals with urinary schistosomiasis. It was therefore not surprising to note that more than half (56.8%) of *S*. *haematobium*-positive participants were mostly dissatisfied or unhappy about their quality of life. Earlier, poor quality of life has similarly been reported among *S*. *haematobium*-positive children and adults [16, 17, 22] and points to the need to intensify efforts to control and eliminate this disease.

The study highlights some important considerations needed for urinary schistosomiasis diagnosis and management. The strengths of the study lie in its relatively representative sample size for the study area and the use of a randomized sampling technique which limits sampling bias. The study should, however, be interpreted considering these limitations: The demographic information collected was not robust enough to explore confounders that may have affected the rate of infection, IPSS, or QOL. The use of a self-reported questionnaire is liable to misinterpretation and social desirability bias. Unfortunately, we were unable to conduct quantitative tests to support participants’ self-rated urinary symptoms. Future studies should consider performing Uroflowmetry or other quantitative assessments of urinary symptoms to further assess the burden of *S*. *haematobium*.

## 5. Conclusion

This study provides important evidence for the inclusion of exercise and water intake in the microscopic diagnosis of *S*. *haematobium*. Again, it highlights that *S*. *haematobium* infection significantly impacts the affected individuals’ urinary health and overall quality of life. These findings reinforce the importance of efforts to control and eradicate this debilitating disease and suggest that multidisciplinary clinical diagnosis and management of schistosomiasis patients be adopted to accurately detect and improve patients’ quality of life. Patients should be made to undertake some exercises and take in water before urine samples are collected for the microscopic examination of ova. Finally, measures should be taken to continuously monitor and manage urinary symptoms in patients with schistosomiasis to improve their quality of life.

## Supporting information

S1 Text The International Prognostic Scoring System (IPSS) questionnaire. (PDF)

S1 Data Raw dataset. (XLSX)
