# Supplementary material for: Toward schistosomiasis control: Assessment of infection-associated voiding symptoms, quality of life and the impact of exercise coupled with water intake on egg recovery in an endemic community in Ghana
Source: PLOS Glob Public Health. 2023 Nov 20;3(11):e0002514. doi: 10.1371/journal.pgph.0002514 (PMC10659178; doi:10.1371/journal.pgph.0002514)
Supplement: S1 Text — (PDF) [file pgph.0002514.s001.pdf]

American Urological Association BPH Symptom Score Index Questionnaire

Having to urinate more frequently, as well as more urgently, can definitely interrupt the flow of your day. You should know that frequent urination is often a symptom of benign prostatic hyperplasia (BPH), a noncancerous enlargement of the prostate gland. BPH is a common condition among men over the age of 50. Waking up several times a night to urinate and having a weaker, slower, or delayed urine stream are other common symptoms.

________________________________ Circle the number that best applies to you

Patient Name **Date**

	not at all	less than 1 time in 5	less than _ the time	about _ the time	more than _ the time	almost always
**1. Incomplete Emptying**<br>Over the last month how, often have you had a sensation of not emptying your bladder completely after you finish urinating?	**0**	**1**	**2**	**3**	**4**	**5**
**2. Frequency**<br>During the last month, how often have you had to urinate again less than two hours after you finished urinating?	**0**	**1**	**2**	**3**	**4**	**5**
**3. Intermittency**<br>During the last month, how often have you stopped and started again several times when you urinate?	**0**	**1**	**2**	**3**	**4**	**5**
**4. Urgency**<br>During the last month, how often have you found it difficult to postpone urination?	**0**	**1**	**2**	**3**	**4**	**5**
**5. Weak Stream**<br>During the last month, how often have you had a weak urinary stream?	**0**	**1**	**2**	**3**	**4**	**5**
**6. Straining**<br>During the last month, how often have you had to push or strain to begin urination?	**0**	**1**	**2**	**3**	**4**	**5**
	None	1 Time	2 Times	3 Times	4 Times	5 Or More Times
**7. Nocturia**<br>During the last month, how many times did you most typically get up to urinate from the time you went to bed until the time you got up in the morning?	**0**	**1**	**2**	**3**	**4**	**5**

Add the score for each number above, and write the total in the space to the right

SYMPTOM SCORE = 1-7 MILD 8-19 MODERATE 20-35 SEVERE TOTAL______

0=Delighted 1=Pleased 2=Mostly Satisfied 3=Mixed 4=Mostly Not Satisfied 5=Unhappy

**8. Quality of life**<br>How would you feel if you had to live with your urinary condition the way it is now, no better, no worse, for the rest of your life.	**0**	**1**	**2**	**3**	**4**	**5**
